# Influence of temperature on embryonic development of *Pontastacus leptodactylus* freshwater crayfish, and characterization of growth and osmoregulation related genes

**DOI:** 10.1186/s40850-024-00198-9

**Published:** 2024-04-28

**Authors:** Maria V. Alvanou, Konstantinos Feidantsis, Athanasios Lattos, Anthi Stoforiadi, Apostolos P. Apostolidis, Basile Michaelidis, Ioannis A. Giantsis

**Affiliations:** 1 Faculty of Agricultural Sciences, University of Western Macedonia, Florina, 53100 Greece; 2 Department of Fisheries & Aquaculture, University of Patras, Messolonghi, 26504 Greece; 3 Laboratory of Ichthyology & Fisheries, Faculty of Agriculture, Forestry and Natural Environment, Aristotle University of Thessaloniki, Thessaloniki, 54124 Greece; 4 Laboratory of Animal Physiology, School of Biology, Faculty of Science, Aristotle University of Thessaloniki, Thessaloniki, 54124 Greece

**Keywords:** *Pontastacus (Astacus) leptodactylus*, Embryonic development, Growth factor, FGFR, Freshwater crayfish, Hatching, Artificial incubation, Elevated temperature, Developmental stages

## Abstract

Narrow clawed crayfish, *Pontastacus (Astacus) leptodactylus*, represents an ecologically and economically valuable freshwater species. Despite the high importance of artificial breeding for conservation purpose and aquaculture potential, hatching protocols have not been developed so far in this species. Further, limited knowledge exists regarding the artificial egg incubation, the temperature effect on embryonic development, hatching synchronization and hatching rate. In the present study we investigated the temperature increase (from 17 ^o^C to 22^o^C) effects in two different embryonic developmental stages of *P. leptodactylus*. Furthermore, two primer pairs for the Fibroblast Growth Factor Receptor 4 (*FGFR4*) gene cDNA amplification were successfully designed, characterising for the first time the FGFR4 gene in *P. leptodactylus* in relation to different developmental stages and temperatures. Apart from the *FGFR4* gene, the *Na+/K+-ATPase α-subunit* expression was also explored. Both the *FGFR4* and *Na+/K+-ATPase α-subunit* expression levels were higher in embryos closer to hatching. Egg incubation at 22^o^C for seven days led to significant increase of *FGFR4* expression in embryos from earlier developmental stages. Nevertheless, temperature increase did not affect *FGFR4* expression in eggs from latter developmental stages and *Na+/K+-ATPase α-subunit* expression in all developmental stages. Temperature increase represents therefore probably a promising strategy for accelerating hatching in freshwater crayfish particularly in early developmental stages. Specifically, our results indicate that *FGFR4* expression increased in embryonic stages closer to hatching and that temperature influences significantly its expression in embryos from earlier developmental stages. Overall, these findings can provide a better understanding of artificial egg incubation of *P. leptodactylus*, and therefore can be employed for the effective management of this species, both for economic and biodiversity retention reasons.

**Supplementary Information:**

The online version contains supplementary material available at 10.1186/s40850-024-00198-9.

## Backround

The narrow-clawed crayfish, *Pontastacus (Astacus) leptodactylus* (Eschscholtz, 1823) represents an ecologically and economically valuable freshwater species which is distributed in European continent [1, 2]. It is characterized by high nutritional value, which leads to high export orientation trade [3]. In general, in the global aquaculture sector crustaceans cover about 9% of production with a continuous annual increase of 10% [4]. Marine shrimp, *Litopenaeus vannamei* dominates the crustacean production with 52%, and it is followed by the freshwater crayfish *Procambarus clarkii* with 21% [5]. Regarding *P. leptodactylus*, Turkey used to be the main export-oriented country from 1970 to 1986 until crayfish plague epidemics devastated species populations with harvests reaching 5.000 tons (in 1984) and then reduced to 200 (in 1991). However, until 2009 crayfish populations recovered reaching 734 tons, translated in 2.713.494 USA dollars [6]. In Greece, there are no such data, however in Lake Polifitou in Northern Greece, crayfish harvest used to be the main income for local fishermen [7] before population devastation [8]. Apart from its nutritional value, it operates as a scavenger, and therefore it possesses a key role towards ecosystem viability [1, 2]. During the last few years, the interest regarding the development of a sustainable aquaculture protocol is increasing, especially in Greece [7]. The urgent need of the development of such a protocol is also highlighted by the mass mortalities events which were recently reported. More specifically, in Vegoritida and Polifitou lakes, a crayfish plague epidemic event occurred and devastated local populations during the last year [8]. Furthermore, apart from economic reasons, biodiversity retention is of main importance because native European crayfish species are vulnerable towards diseases and their population have faced many reductions during the last years [9–12]. The most important pathogen that threaten biodiversity is *Aphanomyces astaci* which cause the crafish plague disease with many outbreaks reported in Europe [13–21] and more recently in Greece as well [8].

One of the first steps for the successful development of an aquaculture protocol is the artificial hatching. Some progress has been made in many freshwater crayfish species towards artificial egg incubation; nevertheless, literature existing on this topic for *P. leptodactylus* is scarce. Previous studies which investigated the development of artificial hatching protocols mainly focused on *Procambarus clarkii* (Girard, 1852) [22] and *Cherax quadricarinatus* (Von Martens, 1868) [23], whereas other studies have been conducted on signal crayfish *Pacifastacus leniusculus* (Dana, 1852) [24, 25], noble crayfish *Astacus astacus* (Linnaeus, 1758) and white-clawed crayfish *Austropotamobius pallipes* (Lereboullet, 1858) [26–30]. Although during the first efforts, the hatchability in artificially incubated eggs was significantly lower in comparison with those attached to the maternal pleopods, the progress in incubation methods, as well as disinfectants, proposed the feasibility of this scenario in *P. clarkii* [22]. Generally, in the most common disinfectants are included formaldehyde and alcohol. Both are useful for successfully control the growth of fungi, kill parasites inactivate most of bacterial species and viruses, and improve hatchability [23, 31–37].

Since, the only available information for *P. leptodactylus* concerns the development of a disinfection protocol for collected eggs [38] and osmoregulation-gene expression [39], artificial incubation of narrow clawed crayfish eggs may provide promising solutions to a plethora of limitations concerning the artificial breeding of this species. Specifically, the establishment of the optimal conditions during hatching may result in higher survival rates, as well as the prevalence of predators and pathogens, e.g. *Aphanomyces astaci* (Schikora, 1906) [30, 40]. Among the advantages of artificial incubation technology are also less energy, space, and labor demand. Furthermore, the successful artificial hatching of healthy crayfish individuals will provide a stock for restocking purposes wherever is needed towards biodiversity retention.

Among the environmental factors that affect growth and development, temperature is pivotal for embryonic development, egg hatching and organism growth [41]. More precisely, brooding success depends mainly on two factors, namely, mean water temperature and egg quality [42]. Temperature is considered one of the most important factors for regulating hypertrophy and hyperplasia in developing embryos [43]. Previous studies have shown that in embryos which hatch possessing a higher muscle fibers number, a higher growth rate regarding the hypertrophic muscle growth pathway is observed [44, 45]. Furthermore, there are some indications that egg incubation temperature can have an impact on muscle growth. For instance, heat incubated eggs of European sea bass *Dicentrarchus labrax* (Linnaeus, 1758) have resulted to significantly improved somatic muscle growth performances [46]. Furthermore, the growth performance may be influenced by incubation temperature prior to hatching in several teleost fish, including Atlantic salmon *Salmo salar* (Linnaeus, 1758) [47–50], halibut *Hippoglossus hippoglossus* (Linnaeus, 1758) [51], haddock *Melanogrammus aeglefinus* (Linnaeus, 1758) [52], pearlfish *Rutilus meidingeri* (Heckel, 1851) [53] and zebrafish *Danio rerio* (F. Hamilton, 1822) [43]. Thus, investigation of the effect of temperature during *P. leptodactylus* embryo development can provide useful information regarding the improvement of growth performance of this slow-growing freshwater crayfish species.

The fibroblast growth factors (FGFs) are a family of polypeptide growth regulators with a significant role in embryonic development. They can stimulate aberrant growth or abnormally affect some aspects of cellular behavior and activate signal transduction pathways leading to diverse biological responses [54, 55]. The function of FGFs takes place in coordination with four highly conserved tyrosine kinase receptors, the fibroblast growth factors receptors (FGFR) [56]. Apart from its substantial role towards embryonic development, FGFR4 is proposed to regulate crayfish innate immunity by modulating NF-κB signaling [57]. Moreover, ATPase enzymes possess a regulatory function towards transport (uptake) of essential ions from aquatic environments. Specifically, in several decapod species, Na^+^/K^+^-ATPase (as a major ion regulator) exhibits a crucial role during the species freshwater environments colonization [58–60]. Hyperosmoregulation functions prior and during hatching, probably initiating during the embryonic phase, allow these organisms to develop entirely in freshwater environments [61, 62].

Overall, the aim of the present study was to investigate the expression of *FGFR* and *Na*^*+*^*/K*^*+*^*-ATPase* genes during embryonic development in artificially incubated *P. leptodactylus* eggs in two different temperatures prior to hatching. Both of these genes were selected for specific reasons:

*FGFR*:

- Possesses a significant role in embryonic development.
- Stimulates normal growth.
- Regulates innate immunity.

*Na*^*+*^*/K*^*+*^*-ATPase*:

- plays a central role in ionoregulation.
- contributes in uptake of essential ions.

The *FGFR* gene was characterized for the first time in *P. leptodactylus*. Since from the above, it is understood that investigation of temperature effect on the *Na*^*+*^*/K*^*+*^*-ATPase* gene expression is of high importance (due to its significant role towards osmoregulation), our intention was to also suggest initial assumptions regarding the effect of incubation temperature on the growth factor and *Na*^*+*^*/K*^*+*^*-ATPase α-subunit* gene expression of *P. leptodactylus* embryos in earlier and latter developmental stages before hatching.

## Materials and methods

### Experimental animals and design

Three berried *P. leptodactylus* females were collected from Vegoritida lake, Greece at end of March 2023 when the water temperature was 14,5 °C. Individuals were acclimated in independent aquaria (90 L, 40 cm long, 50 cm width, 45 cm height) with continuous aeration system to maintain high oxygen levels and constant water temperature at 17 ± 0.5 °C. Fifty eggs were removed from each crayfish by sliding a pair of forceps across the base of the female pleopods from an ovigerous crayfish on 03 April 2023 and transferred into two disinfected 1 L Erlenmeyer flasks equipped with aerated pumps and filled with distilled water. Each flask was covered with parafilm to avoid infection. The temperature at the beginning of the experiment was 17^o^C and the photoperiod was constant 12 h light/12 h dark.

**Fig. 1:**
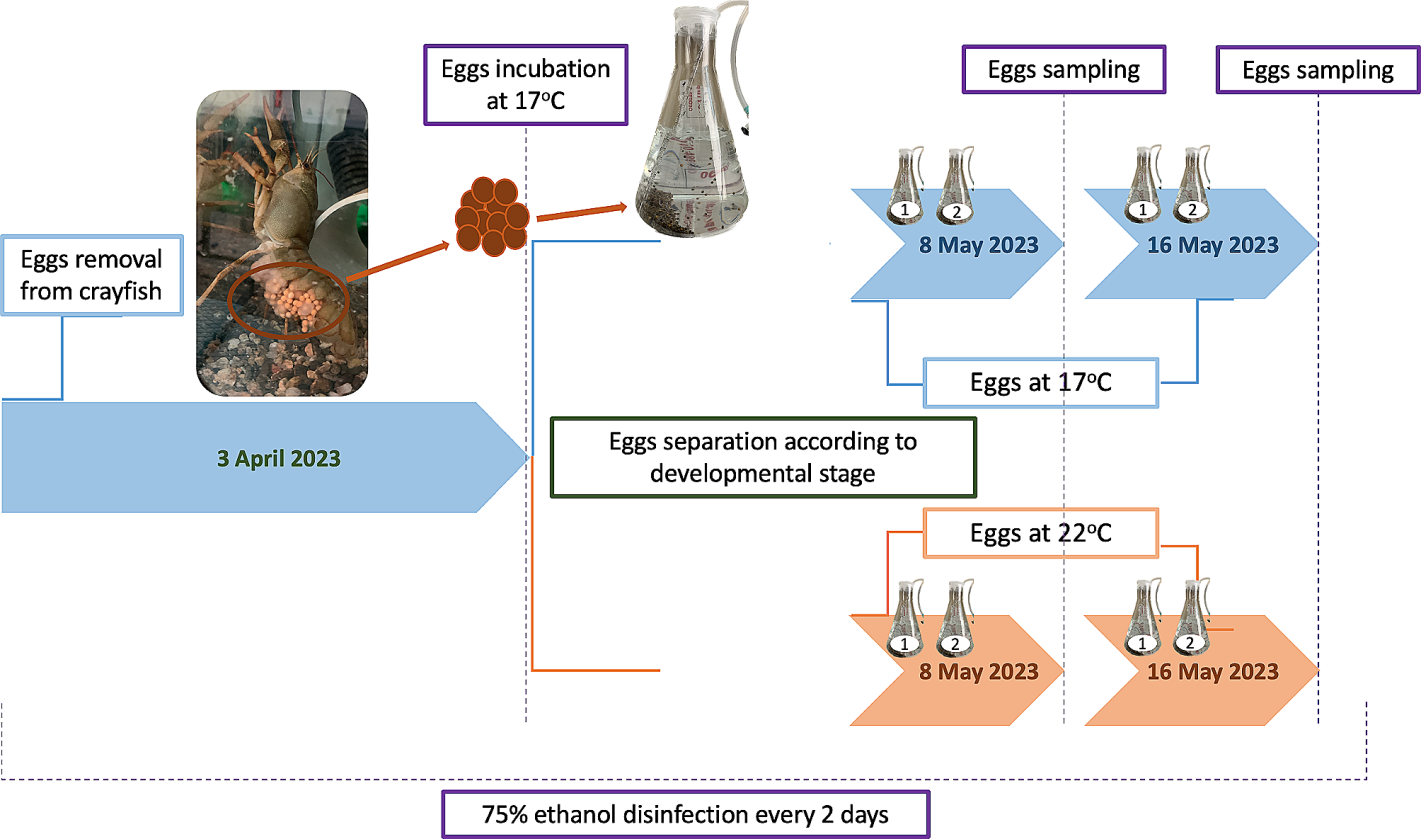
Experimental set-up graphical description

The water was changed every two days and a disinfection process was performed and repeated every two days with 75% ethanol, as previously proposed [23]. This process was repeated until the 8th May 2023, when the development of eggs was observed under the stereoscope. Dead eggs were carefully removed. The developmental stage was determined as described in Sandeman and Sandeman [63]; photos from each developmental stage were examined (Fig. 1). No exact synchronization regarding egg developmental stage was observed; some eggs grew faster. On the 8th May 2023, nine eggs from the 45–55% developmental stage (stage 1) and nine from the 65–85% developmental stage (stage 2) [63] were transferred in disinfected Eppendorf tubes (in groups of three) and kept in -80^o^C until RNA extraction. The remaining eggs were separated in four new disinfected 1 L Erlenmeyer flask. The 2 out of 4 flasks contained eggs from stage 2 while the other two eggs from stage (1) From those containing the eggs from stage 2, the first was kept at 17^ο^C whereas the second one at 22^ο^C. These two temperatures were chosen based on previously published data, as the optimal temperatures for animal growth and increased moulting rate [3, 64–67]. The same experimental design was set for the eggs from stage (2) On the 16th May 2023 nine eggs from each condition were transferred in disinfected Eppendorf tubes (in groups of three) and kept at -80^o^C until the RNA extraction (Fig. 1).

### RNA extraction and cDNA synthesis

Total RNA from each pool containing three eggs was extracted using the NucleoZOL reagent (Macherey-Nagel, Düren Germany), according to the manufacturer’s protocol. From each different developmental stage and treatment. 2.2, each egg from a group of three was pestle-homogenized in 500 µl NucleoZOL, and RNAase-free water was added to the lysate. Three technical replicates from each stage and treatment were performed (*n* = 3 biological replicates, *n* = 3 technical replicates). In the next step, samples were centrifuged, and an iso-propanol solution was added for RNA precipitation. Subsequently, after another one centrifugation, two ethanol washes of the RNA pellet were conducted. Finally, the pellet was resuspended in 100 µl nuclease-free water. The RNA after the extraction was stored at -80^o^C until cDNA synthesis. Prior the reverse transcription (RT), extracted RNA was measured on a Quawell UV-Vis 5000 spectrophotometer (Quawell Technology, San Jose, CA, USA) for the determination of its quantity and quality. cDNA synthesis was performed using the PrimeScript kit (Takara, Japan): approximately 500 ng of total RNA of each sample was used, with the protocol following the manufacturer recommended guidelines. Produced cDNA quality and quantity were also determined in a Quawell UV-Vis 5000 spectrophotometer (Quawell Technology, San Jose, CA, USA). cDNA samples were equally diluted and were then preserved at -20^o^C until qPCR amplification.

### Primer design for FGFR4 gene characterization

For gene expression analysis of the *FGFR4* gene in *P. leptodactylus*, degenerated primer sets were designed according to FGFR4 sequences obtained from NCBI of other crustaceans. Specifically, the sequences with GenBank accession numbers ON012066.1, XM_053780322.1 XM_050877024.1, XM_042383149.1, XM_037929195.1, XM_045266162.1, XM_027376153.1, XM_043018380.1 and ON045327.1 were utilized. The above accession numbers correspond to *P. clarkii*, *C. quadricarinatus* (Von Martens, 1868), *Eriocheir sinensis* (H. Milne-Edwards, 1853), *Homarus americanus* (H. Milne-Edwards, 1853), *Penaeus monodon* (Fabricius, 1798), *Portunus trituberculatus* (Miers, 1876), *Penaeus vannamei* (Boone, 1931), and *Penaeus japonicus* (Spence Bate, 1888) respectively. The sequences were aligned using the MUSCLE algorithm in MEGAX software [68], and the conserved sites were targeted for the design of various primer sets using the Primer 3 software (Primer3_masker, Tartu, Estonia) that were afterwards tested by conventional PCR. Among the tested primers, two sets were eventually selected, the validity of which was confirmed as described in Sect. 2.4.

### Conventional PCR and sequencing

One µl of RT products was utilized as cDNA matrix for amplification in conventional PCR using FastGene Taq 2X Ready Mix (NIPPON Genetics, Duren, Germany) and each one of the newly designed primer sets (Table 1) to test their validity. After the first denaturing step at 95^ο^C for 3 min, 35 cycles of denaturing step for 30 s at 92^ο^C, annealing step for 40 s at 50^ο^C, and extension step for 40 s at 72^ο^C, followed by a final extension step at 72^ο^C for 5 min were performed. In each 20 µL PCR reaction, 0.6 µL of each primer (10 µM), 10 µL FastGene Taq 2X Ready Mix (NIPPON Genetics, Duren, Germany) and 1 µL of cDNA (50 ng/µL) were contained. The volume up to 20 µl was filled with ultrapure water. After the amplification, the PCR products were loaded and run on a 2% agarose for 20 min at 100 V. Subsequently, PCR products were purified using the commercial NucleoSpin Gel and PCR clean up kit (MAcherey-Nagel, Duren, Germany) and were bidirectionally sequenced applying the Sanger methodology. The sequencing results confirmed that the amplified products were the correct parts of the targeted growth factor.

**Table 1:** Primer pairs designed and used for the amplification of the genes targeted in *P. leptodactylus* eggs

Name	Target	Sequence (5’ --> 3’)	Product length (bp)	Reference
FGFR4.1 F FGFR4.1R	FGFR4	5’-ATCATAAACAAGGAGCTGAGT-3’ 5’-TCAACACCATTATASCGWGT-3’	130	Present study
FGFR4.3 F FGFR4.3R	FGFR4	5’-AATGTTCTAGTCAGTGARGA-3’ 5’-CTCTGGAGCCATCCAYTT-3’	128	Present study
NaKF NaKR	Na^+^/K^+^ -ATPase	5’-GGTATGCGAAGTTCCATTT-3’ 5’-TCTCCAAGACCTCCCAGTT-3’	220	[39]
3NaK10F NaK16R	Na^+^/K^+^ -ATPase	5’-ATGACIGTICICAYATGTGG-3’ 5’-GCRTGRTCICCIGTIACCAT-3’	700	[68]
ActF ActR	Actin	5’-CAAGGCYGGYTTCGCYGG-3’ 5’-TCCATRTCRTCCCAGTTGG-3’	200	[39]

### Phylogeny construction

Using the MEGAX software [68], the results from sequencing were aligned using the ClustalW algorithm and a maximum likelihood (ML) phylogenetic tree was created including the sequences with GenBank accession numbers: ON012066.1, XM_053780322.1 XM_050877024.1, XM_042383149.1, XM_037929195.1, XM_045266162.1, XM_027376153.1, XM_043018380.1 and ON045327.1. The sequences were trimmed and only sequences of the same lengths were included in the analysis. For ML tree construction with MEGAX, the best-fit substitution model (K2 + G) was determined applying the Akaike information criterion (AIC). The tree was visualized with MEGAX.

### Gene expression analysis

*FGFR4*, *Na*^*+/*^*K*^*+*^*-ATPase α-subunit* and *actin* gene expression was evaluated through quantitative real time PCR. The comparative CT method (2^–ΔΔCT^ method) as described in Livak and Schmittgen [69] was applied to quantify the relative expression of the two genes during different developmental stages and temperatures of *P. leptodactylus* embryos. The target gene expressions were normalized to actin expression. Real-time quantitative PCRs were carried out in a Thermocycler Eco 48 Real-time PCR (Illumina) instrument using KAPA SYBR® FAST qPCR Master Mix. PCR reactions were performed in 10 µl final volume, where 10 ng of eggs cDNA as template, 5 µl of KAPA SYBR® FAST qPCR Master Mix (2X), 2 µM of each one of the primers (Table 1) and PCR-grade water up to 10 µl were mixed. The thermal profile consisted of an initial step at 95^ο^C for 15 s and 40 cycles of denaturing at 95^ο^C for 15 s, annealing at 55^ο^C for 20 s and elongation at 72^ο^C for 20 s. Plate read was encompassed after the step of 55^ο^C for quantification of the amplicons.

### Statistical analysis

General linear mixed model and repeated measures mixed-model ANOVA (GLM) (independent variables: egg developmental stage, temperature and time) as well as one-way ANOVA were performed to detect significant differences at 5% probability level (SPSS Scientific Inc. Software, version 21). Simple linear correlation (Pearson’s test) analysis was employed for the estimation of significant correlations (at 5% level) between the levels of enzymes of antioxidant defense and apoptotic responses (GraphPad Instat 3.0).

## Results

### Description of the developmental stages

**Fig. 2:**
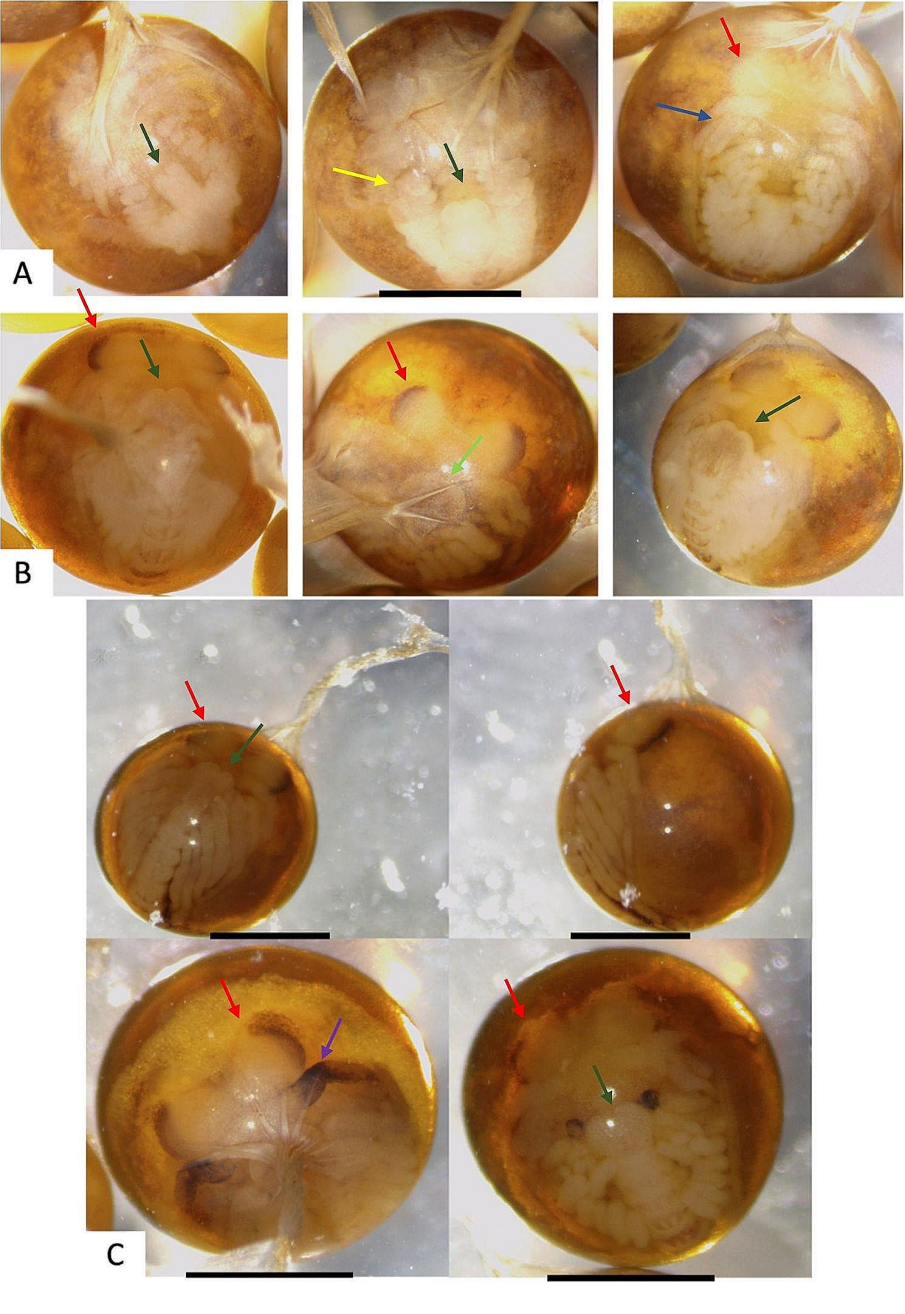
Pictures of *P. leptodactylus* different developmental stages in fixed embryos. (**A**) Developmental stages 45–55%. (**B**) Developmental stages 65–85%. (**C**) Developmental stages 90–95% (Scale bar: 1 mm). Eyes (red arrows), caudal papilla (green arrows), cheliped (purple arrow), Basal joints of antennae and antennules differentiated (blue arrow), mandible (yellow arrow)

The collected eggs were analyzed under the stereoscope and separated according to morphological criteria proposed by Sandeman and Sandeman [63] in *Cherax destructor* (Clark, 1936). Stage 1 eggs presented their eyes pronounced as laterally grown, separated by developing rostrum. Their mandibles were flanked by tips of antennae and their caudal papilla reached the base of the mandibles carapace edge (Fig. 2A). Stage 2 eggs from the later developmental stage possessed differentiated basal joints of antennae and antennules as well as their neural tissue within the eye divided into lobe. Their caudal papilla was covered by appendages tips of the antennae, which reached caudally to the third pair of walking legs (Fig. 2B). Apart from the above stages that were separated in order to conduct the experiment, embryos from 90 to 95% developmental stages were observed under the stereoscope. In these stages the tips of the chelae reached forward to the bases of the eyes and the rostrum grew down between the eyes while the appendages were observed closely packed together and the embryos occupied almost the whole ventral side of the egg (Fig. 2C).

### Phylogeny of the *FGFR4* gene segments

**Fig. 3:**
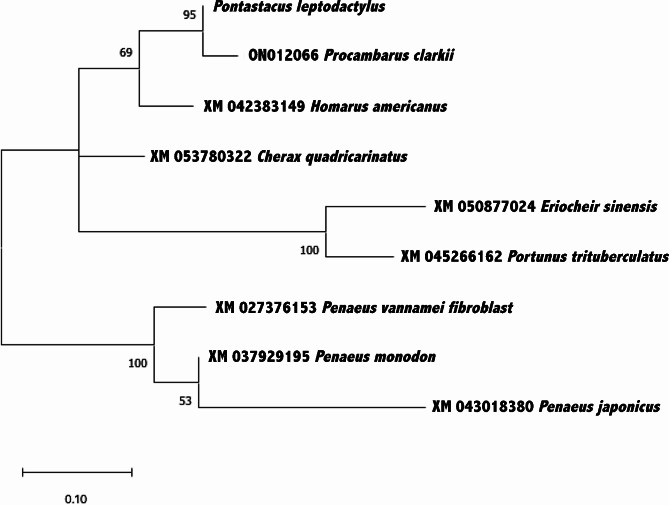
Maximum likelihood dendrogram verifying the validity of the FGFR amplified products. The dendrogram shows the similarity with FGFR gene segments of closely related species, to the greatest extent with *P. clarkii*

The validity of the novel designed primers was evaluated by phylogenetic analysis of the amplified product (Fig. 3). Investigation of the phylogeny of the *P. leptodactylus FGFR4* genotype was based on the alignment of the partial *FGFR4* sequences with the corresponding segments from other haplotypes belonging to eight species within the crustacean subphylum. *P. leptodactylus FGFR4* partial sequence was more closely related to the freshwater crayfish sequences *P. clarkii*. The phylogenetic relationships of *P. leptodactylus FGFR4* partial sequence in comparison with the other species, retrieved from GenBank with accession numbers ON012066.1, XM_053780322.1 XM_050877024.1, XM_042383149.1, XM_037929195.1, XM_045266162.1, XM_027376153.1 and XM_043018380.1 are depicted in the maximum likelihood dendrogram of Fig. 3.

### *FGFR* gene expression

**Fig. 4:**
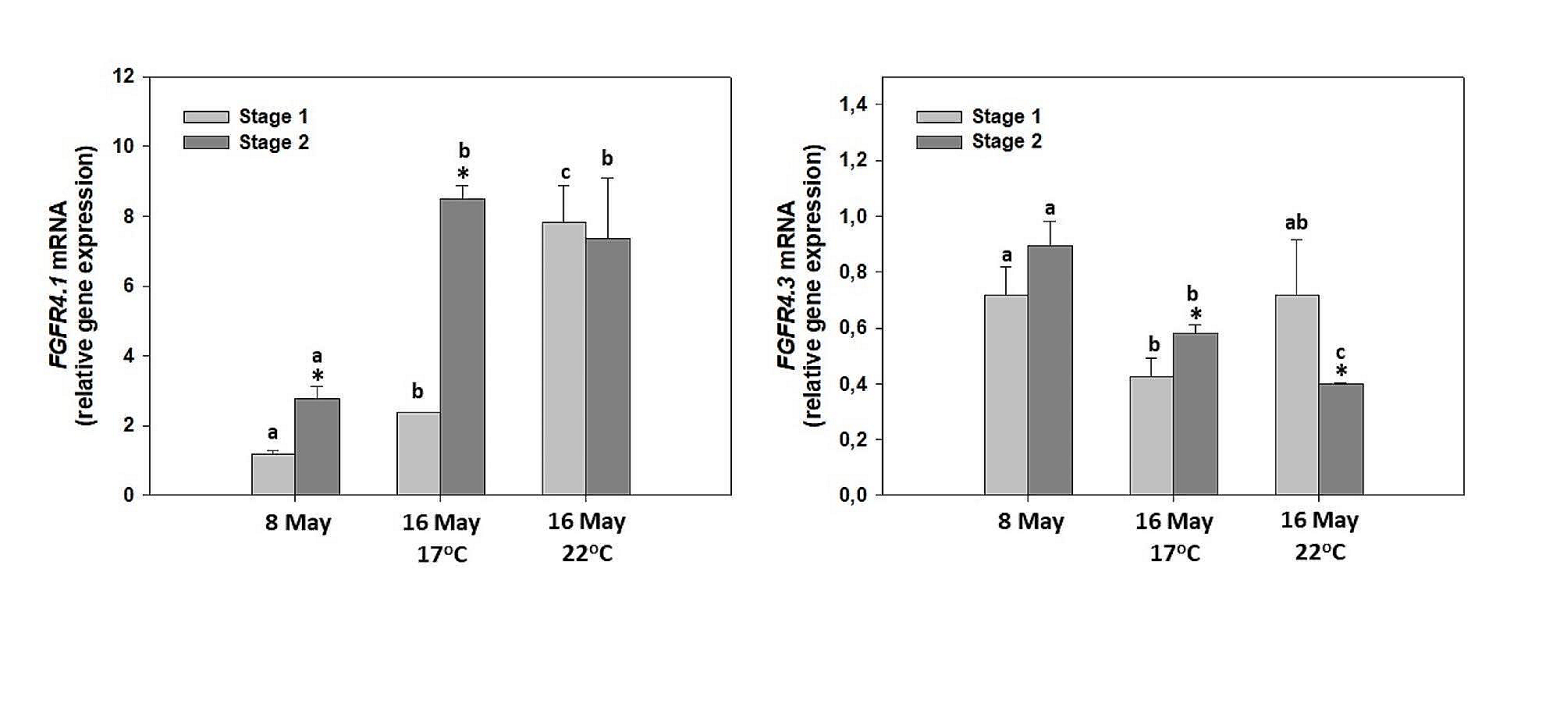
Quantitative PCR analysis of *FGFR4* (parts 1 and 2) mRNA transcripts in *P. leptodactylus* eggs during 2 different developmental stages [stage 1 (45–55%) and stage 2 (65–85%)] using the primer pairs FGFR4.1 and FGFR4.3 in two different time points and two different temperatures prior to hatching. Asterisk (*) denotes statistically significant differences (*p* < 0.05) between stage 1 and stage 2 eggs while lower case letters denote statistically significant differences (*p* < 0.05) between different treatments of the same stage

Expression of *FGFR* mRNA was detected in all examined samples. In general, *FGFR4.1* and *FGFR4.3* amplicons revealed an opposite pattern with *FGFR4.1* increasing with time and temperature, and *FGFR4.3* decreasing (Fig. 4A and B). Specifically, we observed that after the temperature increase in embryos from stage 1, *FGFR4.1* fragment exhibited increased levels of mRNA expression, while such an increase was not observed for stage 2 embryos (Fig. 4A). Regarding *FGFR4.3* fragment, its mRNA expression exhibited decreased levels both over time and with increased temperature for stage 2 eggs, while stage 1 eggs depicted no significant differences (Fig. 4B). In addition, both *FGFR4* parts demonstrated increased levels of mRNA expression in embryos from stage 2 in comparison with those from stage 1, except for 16th of May at 22^o^C where stage 2 eggs revealed no difference compared to stage 1 eggs regarding *FGFR4.1*, while *FGFR4.3* exhibited lower levels in stage 2 compared to stage 1 (Fig. 4A and B).

### *Na*^*+*^*/K*^*+*^*-ATPase* gene expression

**Fig. 5:**
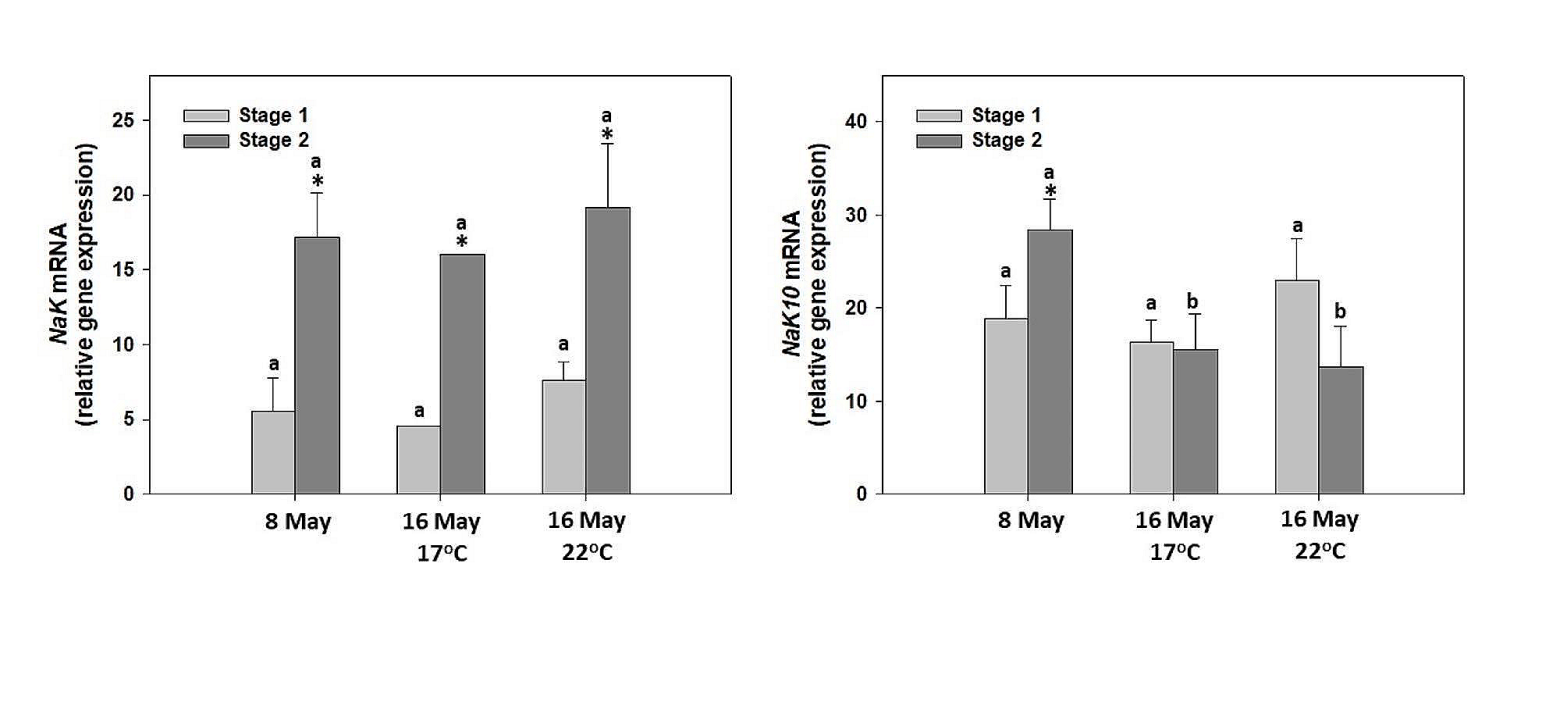
Quantitative PCR analysis of *Na*^*+/*^*K*^*+*^*-ATPase α-subunit* mRNA transcripts in *P. leptodactylus* eggs during 2 different developmental stages [stage 1 (45–55%) and stage 2 (65–85%)] using the primer pairs NaKF/NaKR and NaK10F/NaK16R in two different time points and two different temperatures prior to hatching. Asterisk (*) denotes statistically significant differences (*p* < 0.05) between stage 1 and stage 2 eggs while lower case letters denote statistically significant differences (*p* < 0.05) between different treatments of the same stage

Expression of *Na*^*+/*^*K*^*+*^*-ATPase α-subunit* mRNA was detected in all measured samples with both utilized primer pairs (Table 1). In general, after the amplification of *Na*^*+/*^*K*^*+*^*-ATPase α-subunit* cDNA using primers NaKF/NaKR we observed higher expression levels of the gene in stage 2 eggs (prior to hatching) in comparison with stage 1 eggs (Fig. 5A). On the contrary, after the amplification of *Na*^*+/*^*K*^*+*^*-ATPase α-subunit* cDNA using primers NaK10F/Na16KR we observed higher expression levels of the gene in stage 2 eggs in comparison to stage 1 only before the temperature treatment (Fig. 5B). On the treatments of 16th May, both at 17^o^C and 22^o^C, no significant differences between the two stages were observed. In general, the influence of the water temperature on cDNA expression was not significant (Fig. 5A and B).

### Contribution of variables to biochemical responses

Table 2 exhibits the overall effect of all variables and their interactions. All variables (egg developmental stage, temperature and time) and interactions between variables (egg developmental stage × temperature, and egg developmental stage × time) were statistically significant.

**Table 2:** Results of General Linear Model (GLM) ANOVA analyses

	df	Type III SS	F	*p*
**Egg developmental stage**	3	0.402	6.254	0.007*
**Temperature**	3	0.103	1.766	0.043*
**Time**	3	0.322	4.525	0.018*
**Egg developmental stage x Temperature**	3	0.186	2.244	0.032*
**Egg developmental stage x Time**	3	0.355	5.122	0.011*

*Indicates statistically significant effect

## Discussion

The narrow-clawed crayfish *P. leptodactylus* apart from being a keystone species is a commercially important dietary product. However, a decrease in wild populations is recorded, since the majority of crayfish production is coming from natural stocks existing in the wild. In combination to overfishing, crayfish plague outbreaks synergistically act on this deterioration [8]. It has been reported in previously published studies that non-indigenous European crayfish species outnumbered the indigenous crayfish species in 2:1 ratio, implying that non-indigenous crayfish species can become dominant if the proper measurements towards the protection of indigenous crayfish will not stay in priority [70]. Therefore, from the above, it becomes evident that the development of an aquaculture protocol for these species will be useful towards biodiversity maintenance, as well as, towards local economies. This will mainly occur, as by developing an artificial cultivation protocol bloodstock will be available for further restocking events or for aquaculture applications. The first step towards this direction is to find the optimal conditions for the artificial hatching of the species. However, to the best of our knowledge, scarce data exist regarding the developmental stages of *P. leptodactylus* embryos. Thus, for the first time in *P. leptodactylus* we present the description of embryonic developmental stages, categorized according to Sandeman and Sandeman [63].

Based on the transcriptional levels of the *FGFR4.1* gene, it was observed that in the early developmental stages the expression was increased with temperature increase to 22^o^C, while in the later developmental stages, expression levels increased only with time. On the other hand, the transcriptional levels of the *FGFR4.3* gene decreased both with temperature and time. This unexpected result of the different pattern between the two different primer sets of the same gene (FGFR4) could be explained accordingly: from annotated crustacean genomes and from the limited whole genome sequences available from other freshwater crayfish species, it can be inferred that these organisms possess many large repetitive structures leading to highly challenging assembly and annotation [71, 72]. Thus, entire or partial gene duplication that may have accumulated leading to potential mutation in the one of the two parts of the gene cannot be excluded, making the expression pattern of this gene inconsistent. The present results also demonstrate the fact that *P. leptodactylus* egg hatching was accelerated by the temperature increase from 17^o^C to 22^o^C; the latter is also in general reflected in the FGFR gene expression. In line with our results, and in line with the aforementioned studies, Jin et al. (2019) proposed 25^o^C as the optimal temperature for embryonic development in the freshwater crayfish species *P. clarkii.* Specifically, the hatching period was shortened, and the embryonic development was accelerated parallel to water temperature increase in a suitable range of 17-25^o^C [73–75]. The results were surprising in the study of Jin et al. [73], as the hatching period in 25^o^C was reduced in one fourth (21 days) in comparison to 85 days at 17^o^C. A similar pattern was also observed in other crustaceans concerning the influence of the water temperature on hatching timing and embryonic development [76–81].

Similarly, to the crustaceans, incubation temperature seems to influence the timing of teleost fish eggs’ hatching. Apart from hatching timing, incubation temperature is an environmental factor, which affects fish growth and muscle fibers composition [29]. Specifically, in the teleost fish species *Anabas testudineusis* (Bloch, 1792), temperature increase to 28^o^C prior to hatching leads to alterations in the formation of hyperplastic muscle fibers and induction of gene expression-related to growth factors [82]. In the same species, the incubation temperature of 28^o^C resulted to increased hatching rates and reduced the number of abnormal and deformed larvae [83]. Ιn the Russian sturgeon *Acipenser gueldenstaedtii* (von Brandt & Ratzeburg 1833) the hatching rate was increased in correspondence to a temperature increase from 12^o^C and 16^o^C to 20^o^C [84]. Generally, it has been observed that the variability in eggs incubation temperature even in low ranges can lead to substantial changes on the development of embryonic stages [85–87]. Hence, as proposed by Güralp et al. [88], the development and timing of embryonic development and hatching apart from closely related to temperature, is species specific as well.

Although temperature increase exhibited a significant effect on the FGFR gene expression levels, it seemed to not significantly influence *Na*^*+/*^*K*^*+*^*-ATPase α-subunit* gene expression levels in the present study. To our knowledge, no literature exists on the effect of temperature on the Na^+/^K^+^-ATPase function during embryonic development in crayfish. However, some data exist regarding fish species. Similar to our results, the Na-K pump activity in perch *Perca fluviatilis* (Linnaeus, 1758) and ruffe *Gymnocephalus cernuus* (Linnaeus, 1758) embryos clearly shows that the most favorable temperatures for early embryonic development are its lower values, while after hatching, increased temperature increases the activity of this enzyme. However, in roach *Rutilus rutilus* (Linnaeus, 1758), the optimum water temperature for early embryonic development is in general high, but the latter corresponds to the fact that this species is a more thermophilic one compared to perch and ruffe [89]. Therefore, we can assume, taking into consideration *P. leptodactylus* biology, that our investigated species is more similar regarding its embryonic development to fish species such as perch and ruffe, than to more thermophilic ones. Contrary to the non-temperature effect, a 3-fold increase was observed in *Na*^*+/*^*K*^*+*^*-ATPase α-subunit* expression in stage 2 eggs compared to stage 1. In line with our results Serrano et al. [39] observed a peak in *Na*^*+/*^*K*^*+*^*-ATPase α-subunit* gene expression levels in *P. leptodactylus* embryos prior to hatching. The increasing activation and transcription of one of the main ion transporter genes toward the end of the embryonic development of *A. leptodactylus* contributes to the acquisition of efficient hyperosmoregulation, which is used at hatch when the emerging juvenile faces the sudden osmotic stress originating from exposure to freshwater [39, 61]. Unlike marine and brackish species larvae, in which the ability to osmoregulate generally occurs during the metamorphic transition, the freshly hatched crayfish juveniles and not the eggs must possess physiological mechanisms to cope with the osmotic stress linked to the freshwater environment (massive water influx and ion loss) [61]. Nevertheless, the present study’s results exhibited that the expression pattern wasn’t the same for the second primer pair for Na^+/^K^+^-ATPase (NaK10F/Na16KR). This could be probably attributed to the bigger length of the targeted fragment (700 bp) which makes it less appropriate for Na^+/^K^+^-ATPase expression studies, in comparison with the other fragment occurred from first primer pair (NaKF/NaKR), corresponding to 220 bp. However, it should be underlined that studies dealing with Na^+/^K^+^-ATPase during the embryonic phase of crustaceans are very scarce and mostly dedicated to marine and estuarine decapod species [90].

## Conclusions

To conclude, in the present study we designed two primer pairs for the *FGFR4* gene amplification and therefore characterized its expression in *P. leptodactylus* embryos of differential stages for the first time. We partially sequenced *FGFR4* gene and assessed the influence of the temperature increase towards its expression levels during *P. leptodactylus* embryonic development. From the obtained results we observed significant increase of *FGFR4* in later developmental stages in comparison with the earlier ones. Furthermore, an increase in water temperature by 5^o^C (from 17^o^C to 22^o^C) increased *FGFR4* expression mostly in embryos from early developmental stages, therefore implying *P. leptodactylus* growth rate acceleration leading to hatching. Regarding *Na*^*+/*^*K*^*+*^*-ATPase* gene expression, it generally remained unaffected by the temperature increase. Combined together, the above-mentioned inferences indicate that although the increase in water temperature can increase the expression levels of the *FGFR* gene related to embryonic growth, which is also implicated in innate immunity of the crayfish, the *Na*^*+/*^*K*^*+*^*-ATPase* gene involved in osmoregulation remains unaffected. The temperature increase combined with growth factors expression increase represent a promising strategy for hatching acceleration and cellular muscle growth increase, especially in early developmental embryonic stages. Therefore, this study aimed to investigate whether different incubation temperature of eggs prior to hatching can stimulate growth-related gene expression in their early and late developmental stages. The data produced herein, could operate as a first step towards the understanding of the water temperature influence in the artificial incubation of *P. leptodactylus* eggs and as a result in the development of laboratory reproduction and development protocols, mainly by influence the hatching and growth rate while keep unaffected the osmoregulation processes. The development of an artificial hatching protocol can contribute to the optimization of an aquaculture protocol contributing to biodiversity maintenance and food production.

### Electronic supplementary material

Below is the link to the electronic supplementary material.

Supplementary Material 1: *Pontastacus leptodactylus* embryos at the stage of beating heart indicative of the heart beat.

## Data Availability

The sequence generated and analysed during the current study is available in the GenBank database, [ACCESSION NUMBER: PP575821].
